# Lagophthalmos of the upper eyelid after rhinoplasty: A case report

**DOI:** 10.1002/ccr3.4776

**Published:** 2021-09-16

**Authors:** Hesam Jahandideh, Fatemeh Dehghani Firouzabadi, Mohammad Dehghani Firouzabadi, Maryam Roomiani

**Affiliations:** ^1^ ENT and Head & Neck Research Center The Five Senses Health Institute Iran University of Medical Sciences Tehran Iran; ^2^ Department of Otolaryngology‐Head and Neck Surgery Firoozgar Hospital Iran University of Medical Sciences Tehran Iran

**Keywords:** adverse effect, case report, lagophthalmos, rhinoplasty, upper eyelid

## Abstract

Lagophthalmos is an inability to close the eyelids which can result from many causes. Septorhinoplasty surgery is an uncommon reason for that. This paper reports the lagophthalmos complication, after a septorhinoplasty surgery.

## Graphical abstract

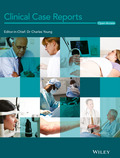

## 1 INTRODUCTION

Lagophthalmos is an inability to close the eyelids, which can yield to high tear evaporation, corneal abrasion, and keratopathy, as well as cosmetic issues and their consequent psychological concerns. This paper presents a 28‐year‐old woman patient underwent a primary septorhinoplasty in a private clinic, to improve her nasal form and function. The patient presents with left upper eyelid lagophthalmos in her follow‐up visit and complete resolution occurred following three weeks of conservative treatment.

Lagophthalmos is defined as the inability to close the eyelids completely, which can lead to a corneal abrasion due to the evaporation of the tear and unprotected eye.^1^, ^2^ A variety of etiologies has been reported for lagophthalmos, mainly related to facial nerve damage. From infectious diseases, such as otitis or mastoiditis, to various facial nerve‐related tumors, traumas, toxins (such as thalidomide or diphtheria), and iatrogenic etiologies such as facial surgeries, embolization, or dental surgeries.^3,4^ The primary goal of managing the issue is stopping the exposure keratopathy, which may have serious consequences including blindness. Cosmetic issues should also be considered in the management of the condition.^5^

Several post rhinoplasty complications are reported, among them, infection, septal perforation, airway obstruction, scar formation, numbness, or cosmetic dissatisfaction are very well known.^6^, ^7^ Lagophthalmos is unusual after septoplasty while it is common as a result of temporofrontal branch injury, excessive tissue resection in patients undergoing simultaneous forehead/brow procedures and upper lid blepharoplasty.^1^ This paper reports a rare case with lagophthalmos, followed by a septorhinoplasty surgery performed to correct the nasal form and function.

## 2 CASE REPORT

A 28‐year‐old woman patient was admitted to the private clinic with the complaint of deformity of nose and breathing problem. The physical examination of the patient showed septal deviation. The endoscopic evaluation of the patient was otherwise normal. Her paranasal sinus CT scan also confirmed septal deviation without any further pathologies (Video S1).

Surgical procedures included septoplasty, tip plasty, and bilateral internal lateral osteotomies with an open approach. General anesthesia without complication was performed for the patient. Bilateral lateral osteotomies were done to correct the deviated external nose.

Incomplete left upper eyelid closure was detected in the first postoperative visit 2 days after surgery (Video S2A). A paralysis was localized in the left upper eyelid. Ophthalmology consultation confirmed the left upper eyelid lagophthalmos with corneal erosion.

Eyedrops and night‐time patching were prescribed for the patient. After 21 days, the eyelid closure was improved and the patient was able to close her eyelids completely (Video S2B). No evidence of recurrence or any other complications was observed during her longer follow‐up till 6 months.

## 3 DISCUSSION

This paper reports the lagophthalmos complication, after a septorhinoplasty surgery. There are several complications, mentioned in the literature for rhinoplasty surgery.^6^, ^8^, ^9^ First, early serious complications such as hemorrhage, infection, and skin or septal necrosis. Second, skin complaints such as acne, scar formation, bruising, or erythema, and unsatisfying outcomes as the third category of the complications. However, neurological complications of this type of surgery, ranging from sensory deficits in the area of the nose to muscular dysfunctions due to motor neural deficits are less investigated.

Our search in the literature found only one article about lagophthalmos following septorhinoplasty. The authors have concluded that this complication is due to external osteotomy incision and upper eyelid hematoma and facial nerve neurapraxia as a result of hematoma pressure.^2^ But according to the typical appearance of our patient presentation, we are believed that the direct nerve injury is better explanation for this finding.

It is noted that considering variations of the regional anatomy is important to prevent surgical adverse effects in each patient.^8^ While according to the references, most of the surgeons think that the upper orbicularis oculi muscle innervated by temporal branch of facial nerve,^10^ there is conflicting evidence about the innervation of orbicularis oculi muscle fibers in the upper eyelid region as well as the course of buccal branch of the facial nerve.^11^, ^12^ According to these precise anatomical studies the terminal fibers of buccal branch of facial nerve, coursing from lateral parts of the bony pyramid of nose are responsible for innervating the medial part of upper eyelid orbicularis muscle. The course of these fibers is compatible with lateral nasal osteotomy site. So, the indirect damage of nerve fibers in terms of neurapraxia may be better explanation for temporary lagophthalmos.

Due to the suspected nerve damage, our patient followed up for three weeks after surgery. The patient's symptoms alleviated in 21 days and became symptom‐free in that period. Lubricant drops and eye patching were prescribed as conservative therapies for 3 weeks to control the corneal abrasion.

There are two main approaches in lagophthalmos treatment: clinical therapy and surgical therapy. The clinical therapy consists of using artificial tears, ointments, or nighttime‐eye patching to restore the tear film and reduce the corneal abrasion. The botulinum toxin injection is also suggested to reduce the lid levator muscles tone.^1^, ^3^

This very rare unknown complication of rhinoplasty may be very frightening for the surgeon. Increasing familiarity with these types of problems may have a great role in reassuring the surgeon and the patient. Also revising and updating anatomical knowledge as a must for any surgeon could not be more overemphasized in the shade of these kinds of rare but fearsome complications.

## CONFLICT OF INTEREST

Authors declare no conflict of interest.

## AUTHOR CONTRIBUTIONS

HJ and MR conceptualized and designed the work. HJ, FDF, MDF, and MR drafted the article. HJ, FDF, and MDF critically revised the article. All the authors approved the final version and have the agreement to be accountable for all aspects of the work in ensuring that questions related to the accuracy or integrity of any part of the work are appropriately investigated and resolved.

## ETHICS APPROVAL

This study protocol was approved by the local ethics committee of the Iran University of Medical Sciences. Informed consent was obtained from the patient before the study.

## Supporting information

Video S1

Video S2

## Data Availability

All data generated or analyzed during this study are included in this submitted article.
